# β-Elemene-induced autophagy protects human gastric cancer cells from undergoing apoptosis

**DOI:** 10.1186/1471-2407-11-183

**Published:** 2011-05-20

**Authors:** Jing Liu, Ye Zhang, Jinglei Qu, Ling Xu, Kezuo Hou, Jingdong Zhang, Xiujuan Qu, Yunpeng Liu

**Affiliations:** 1 Department of Medical Oncology, the First Hospital of China Medical University, Shenyang 110001, P.R.China

## Abstract

**Background:**

β-Elemene, a compound found in an herb used in traditional Chinese medicine, has shown promising anti-cancer effects against a broad spectrum of tumors. The mechanism by which β-elemene kills cells remains unclear. The aim of the present study is to investigate the anti-tumor effect of β-elemene on human gastric cancer cells and the molecular mechanism involved.

**Results:**

β-Elemene inhibited the viability of human gastric cancer MGC803 and SGC7901 cells in a dose-dependent manner. The suppression of cell viability was due to the induction of apoptosis. A robust autophagy was observed in the cells treated with β-elemene; it was characterized by the increase of punctate LC3 dots, the cellular morphology, and the increased levels of LC3-II protein. Further study showed that β-elemene treatment up-regulated Atg5-Atg12 conjugated protein but had little effect on other autophagy-related proteins. PI3K/Akt/mTOR/p70S6K1 activity was inhibited by β-elemene. Knockdown of Beclin 1 with small interfering RNA, or co-treatment with the autophagy inhibitor, 3-methyladenine or chlorochine enhanced significantly the antitumor effects of β-elemene.

**Conclusions:**

Our data provides the first evidence that β-elemene induces protective autophagy and prevents human gastric cancer cells from undergoing apoptosis. A combination of β-elemene with autophagy inhibitor might thus be a useful therapeutic option for advanced gastric cancer.

## Background

Gastric cancer is one of the top three leading causes of cancer death in China. Most patients present with advanced disease which limits their surgical options. Chemotherapy is thus the major treatment for advanced gastric cancer, but the outcome is still very poor, with a median overall survival time of less than 1 year [1]. Combined chemotherapy with cytotoxic drugs usually leads to severe toxicity which lowers the quality of life of patients. Thus, new agents with high anti-tumor activity but low side effects are urgently needed.

Elemene (1-methyl-1-vinyl-2,4-diisopropenyl-cyclohexane) is a novel lipid-soluble anticancer drug extracted from the traditional Chinese medicinal herb *Rhizoma zedoariae *[2]. β-Elemene, the active component of elemene, has been shown to be effective against various tumors such as lung cancer, colorectal cancer and glioblastoma [3-5]. In China, β-elemene has been used to effectively treat certain types of tumors in the clinic, and it presents fewer side effects than other cytotoxic agents [6,7]. However, the mechanisms by which β-elemene kills cancer cells are still not clear. Recent studies showed that β-elemene inhibited cell proliferation by inducing apoptosis as well as cell cycle arrest [3,8]. Others reported that the apoptosis triggered by β-elemene was through the mitochondrial-mediated pathway, as it was accompanied by a reduction of Bcl-2, Bcl-X(L) and XIAP [4,9]. Yet the exact mechanisms still need to be further elucidated.

Autophagy is an intracellular degradation system that delivers cytoplasmic constituents to the lysosome [10]. Under normal conditions, autophagy is a mechanism for the turnover of proteins and elimination of damaged organelles to maintain cell homeostasis [11]. It starts with the formation of double membrane vesicles (autophagosomes) which engulf organelles or long-lived proteins. The autophagosomes then fuse with lysosomes, forming the autophagolysosome, in which the contents are degraded [10,11]. It has been suggested that autophagy induced under pathological conditions functions as an adaptive cell response, allowing the cell to survive bioenergetic stress [12]. However, extensive or persistent autophagy also results in cell death [13]. Thus, autophagy is an important and decisive factor in the balance between cell death and survival.

Recent studies have shown that some chemotherapeutics known to activate apoptosis also induce autophagy [14]. Inhibition of autophagy by pharmacological inhibitors can enhance the anti-tumor activity of cytotoxic agents [15,16]. In these cases, autophagy serves as a protector - it prevents cells from undergoing apoptosis. However, autophagy can also do the opposite; it can kill cells by inducing autophagic cell death [17,18]. The molecular mechanisms by which autophagy regulates survival and death need to be further studied.

In the present study, we report that β-elemene induces apoptosis as well as protective autophagy in human gastric cancer cells. Induction of autophagy was associated with inhibition of the PI3K/Akt/mTOR signaling pathway, and inhibition of autophagy could enhance β-elemene-induced apoptosis.

## Methods

### Cell cultures

The human gastric cancer cells MGC803 and SGC7901 were obtained from the Type Culture Collection of the Chinese Academy of Sciences (Shanghai, China). The cells were cultured in RPMI-1640 medium (Gibco) containing 10% heat-inactivated fetal bovine serum (FBS), penicillin (100 U/mL) and streptomycin (100 mg/mL) at 37°C under an atmosphere of 95% air and 5% CO2. The cells were routinely subcultured every 2-3 days, and were all from the logarithmic phase of growth.

### Reagents and antibodies

β-Elemene was obtained from Yuanda Pharmaceuticals (Dalian, China). 3-Methyladenine (3-MA) and chlorochine (CQ) were purchased from Sigma-Aldrich (St. Louis, Mo. USA). LysoTracker and Hoechst33342 were from Invitrogen (USA). Anti-Bcl-2, anti-Bax, anti-Survivin, anti-actin and anti-Akt antibodies were purchased from Santa Cruz Biotechnology (USA). Anti-LC3, anti-Beclin 1, anti-Atg5, anti-Atg9 and anti-Atg16L antibodies were from Novus Biological (Littleton, CO, USA). Anti-caspase 3, anti-poly(ADP-ribose) polymerase (PARP), anti-phospho-Akt (Ser-473), anti-phospho-mTOR, anti-mTOR, anti-phospho-p70S6K1, and anti-p70S6K1 antibodies were purchased from Cell Signaling Technology (USA).

### Cell viability assay

Cell viability was measured using a 3-(4, 5-dimethylthiazol-2-yl)-2, 5-diphenyltetrazolium bromide (MTT) assay. The cells were seeded at 5 × 10^4 ^cells/well in 96-well plates, incubated overnight and then exposed to the indicated concentrations of β-elemene for the indicated times. Thereafter, 20 μl of MTT solution (5 mg/mL) was added to each well, and the cells were incubated for another 4 h at 37°C. After removal of the culture medium, the cells were lysed in 200 μl of dimethylsulfoxide (DMSO), and the optical density (OD) was measured at 570 nm with a microplate reader (Model 550; Bio-Rad Laboratories, USA). The following formula was used: cell viability = (OD of the experimental sample/OD of the control group) × 100%.

### Analysis of apoptosis

Cells were seeded at 3 × 10^5 ^cells/well in 6-well plates, incubated overnight and then exposed to the indicated concentrations of β-elemene for the indicated times. Cells were collected and incubated with 1 μg/mL Annexin V for 20 min in the dark. Finally, the samples were evaluated by flow cytometry and the data were analyzed using WinMDI software.

### Fluorescence microscopy

For the analysis of green fluorescent protein-fused LC3 (GFP -LC3) localization, MGC 803 cells were transfected with a plasmid encoding GFP-LC3 (kindly provided by Høyer-Hansen M, Danish Cancer Society) and stably expressing cells were selected with changes of media containing 200 μg/mL of G418. Transfection was performed using Lipofectamine 2000 reagent (Invitrogen, USA), according to the manufacturer's instructions. After treatment of 10 or 50 μg/mL β-elemene for 24 h, cells were incubated with 50 nmol/L LysoTracker for 30 min at room temperature, and the nucleus was stained with Hoechst33342. The images were obtained with a fluorescence microscope (Olympus, Japan). The detection of punctated GFP-LC3 co-locolized with LysoTracker indicated the formation of autophagosomes.

### Transmission electron microscopy

Cells were treated and collected by trypsinization, then fixed with 2.5% phosphate-buffered gluteraldehyde, postfixed in 1% phosphate-buffered osmium tetroxide. Cells were then embedded, sectioned, double stained with uranyl acetate and lead citrate, and analyzed using a JEM-1200EX transmission electron microscope (JEOL, Japan).

### Western blotting

Cells were washed twice with ice-cold PBS and solubilized in 1% Triton lysis buffer [1% Triton X-100, 50 mmol/L Tris-Cl (pH 7.4), 150 mmol/L NaCl, 10 mmol/L EDTA, 100 mmol/L NaF, 1 mmol/L Na_3_VO_4_, 1 mmol/L PMSF and 2 μg/mL aprotinin] on ice, then quantified using the Lowry method. Cell lysate proteins (50 μg) were separated by sodium dodecyl sulfate-polyacrylamide gel electrophoresis and electrophoretically transferred to nitrocellulose membranes (Immoblin-P; Millipore, USA). The membranes were blocked with 5% skim milk in TBST buffer [10 mmol/L Tris (pH 7.4), 150 mmol/L NaCl and 0.1% Tween-20] at room temperature for 1 h and incubated overnight at 4°C with the indicated primary antibodies. After the membranes were washed with TBST buffer, they were reacted with the appropriate horseradish peroxidase-conjugated secondary antibodies for 30 min at room temperature. After extensive washing with TBST buffer, the proteins were visualized with enhanced chemiluminescence reagent (SuperSignal Western Pico Chemiluminescent Substrate; Pierce, Rockford, IL, USA). The images were analyzed using NIH Image J software.

### Clonogenic assay

Cells were seeded at 5 × 10^4^cells/well in 12-well plates and treated with either 50 μg/mL β-elemene or 20 μmol/L CQ, or co-treatment with β-elemene and CQ for 6 h. Cells were then plated (in triplicate) at 200 cells per well into 12-well plates with fresh drug-free medium. Cells were incubated for an additional 14 days, and the clones in each well were stained, counted and photographed.

### RNA interference

Small interfering RNA corresponding to the human Beclin 1 cDNA sequence (5'-CAGTTTGGCACAATCAATAtt-3') [19] and a control siRNA (5'-UUCUCCGAACGUGUCACGUtt-3') were from Genechem Co. (Shanghai, China). Cells were transfected with either Beclin 1 siRNA or control siRNA at 100 nmol/L using DharmaFECT transfection agent (Dharmacon Research, CO, USA) according to the manufacturer's guidelines. Forty-eight hours after transfection, the cells were subcultured for further use. The expression of Beclin 1 was verified by western blotting.

### Statistical analysis

The experiments were repeated at least three times. Data are expressed as the means ± SD. Differences in the results for two groups were evaluated by the Student's t-test. P < 0.05 was considered to be statistically significant.

## Results

### β-Elemene inhibited cell viability and induced apoptosis

**Figure 1:**
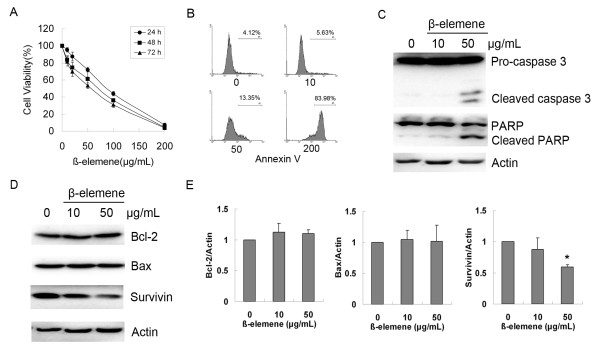
**Effects of β-elemene on cell viability and apoptosis of MGC803 cells**. (A) MGC803 cells were treated with β-elemene at the indicated concentrations for 24, 48 or 72 h, and the cell viability was analyzed by MTT assay. Dots: mean of three independent experiments; bars: SD. (B) MGC803 cells were treated with 10, 50 and 200 μg/mL β-elemene for 24 h, and apoptosis was determined by flow cytometry followed by Annexin V staining. (C) Cells were treated with either 10 or 50 μg/mL β-elemene for 24 h, and the cleavage of caspase 3 and PARP was detected by western blotting. (D) Cells were treated as described above, and the expression of Bcl-2, Bax and Survivin was detected by western blotting. The results were representatives of three independent experiments. Actin was used as loading control. (E) The levels of Bcl-2, Bax and Survivin were measured by NIH Image J software and corrected for actin. Columns: mean of three independent experiments; bars: SD. * *P*<0.05 *vs*. untreated control.

Human gastric cancer MGC803 cells were treated with β-elemene at concentrations ranging from 10 μg/mL to 200 μg/mL for 24, 48 or 72 h. Cell viability assays showed that β-elemene inhibited cell growth in a dose-dependent manner (Figure 1A). The IC_50 _values of β-elemene at 24, 48 and 72 h were 80.03 μg/mL, 56.03 μg/mL and 45.05 μg/mL, respectively. Flow cytometry assays showed a significant increase in the apoptotic population among the cells treated with β-elemene at 24 h (Figure 1B). To further confirm that apoptosis was induced by β-elemene, western blotting was performed to detect the cleavage of caspase 3 as well as PARP. As shown in Figure 1C β-elemene clearly cleaved pro-caspase 3 and PARP to their active forms. The effects of β-elemene on the expression of apoptosis associated proteins were further investigated. Western blotting showed that β-elemene had little effect on the expression of Bax or Bcl-2, but significantly down-regulated the level of Survivin (Figure 1D and 1E). These data indicated that β-elemene inhibits cell viability through inducing apoptosis in human gastric cancer cells.

### β-Elemene induced autophagosome formation

**Figure 2:**
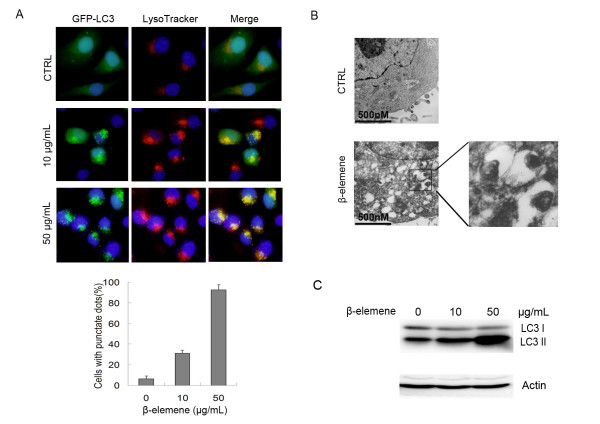
**β-Elemene induced autophagy in MGC803 cells**. (A) MGC803 cells stably expressing GFP-LC3 were treated with either 10 or 50 μg/mL β-elemene for 24 h, stained and observed under a fluorescence microscope as described in Materials and Methods. The percentage of cells with punctuate dots was represented with a histogram. Columns; mean of three independent experiments, bars: SD. (B) Cells were treated with 50 μg/mL β-elemene for 24 h, then harvested and subjected to transmission electron microscopy as described in Materials and Methods. (C) After the cells were exposed to 10 or 50 μg/mL β-elemene for 24 h, cell lysates were subjected to western blotting with an anti-LC3 antibody. The results were representatives of three independent experiments. Actin was used as loading control.

It has been reported that some lipid-soluble anti-tumor agents such as oleandrin could simultaneously induce apoptosis and autophagy [20], so we asked if there was any autophagy in the cells treated with β-elemene. MGC803 cells stably expressing GFP-LC3 were treated with 10 or 50 μg/mL β-elemene for 24 h, and the localization of GFP-LC3 was evaluated under fluorescent microscopy. As shown in Figure 2A, only a few LC3-positive puncta were observed in untreated control cells. However, in the cells treated with 10 μg/mL β-elemene, over 30% of cells were observed with LC3-positive puncta, and in the cells treated with 50 μg/mL β-elemene, more than 90% of cells showed LC3-positive puncta. GFP-LC3 co-localized with LysoTracker after treatment with β-elemene, which suggests the accumulation of autophagosomes. The formation of autophagosomes was further confirmed by transmission electron microscopy. Upon treatment of 50 μg/mL β-elemene many autophagic vesicles, double membrane enclosed vesicle containing engulfed organelles, were observed in the cytoplasm (Figure 2B). Meanwhile, β-elemene treatment also significantly increased LC3-II levels as demonstrated by western blotting (Figure 2C). These data indicate that β-elemene treatment not only results in apoptosis, but also induces autophagy.

### Effects of β-elemene on autophagy-associated proteins

**Figure 3:**
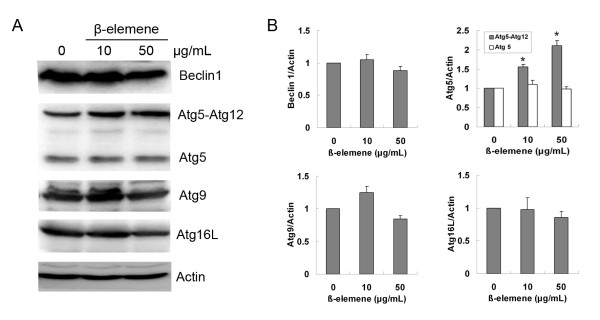
**Effect of β-elemene on the expression of autophagy-related proteins**. (A) After the cells were exposed to 10 or 50 μg/mL β-elemene for 24 h, cell lysates were subjected to western blotting with specific antibodies. The results were representatives of three independent experiments. Actin was used as loading control. (B) The levels of Atg proteins were measured by NIH Image J software and corrected for actin. Columns; mean of three independent experiments, bars: SD. * *P*<0.05 *vs*. untreated control.

Autophagy is regulated by a group of proteins called Atg (autophagy-related) proteins [10]. Some stimuli can modulate autophagy simply by changing the expression of certain Atg protein [21]. To determine if β-elemene could affect the expression of Atg proteins, MGC803 cells were treated with 50 μg/mL β-elemene for 24 h and the levels of some Atg proteins were detected by western blotting. After treatment with β-elemene, the level of Atg5-Atg12 conjugated protein was up-regulated, whereas the expression of Beclin1, Atg5, Atg9 and Atg16L was not affected significantly (Figure 3A and 3B). These data suggest that the up-regulation of Atg5-Atg12 conjugated protein might contribute to the induction of autophagy by β-elemene.

### β-elemene inhibited PI3K/Akt/mTOR signalling pathway

**Figure 4:**
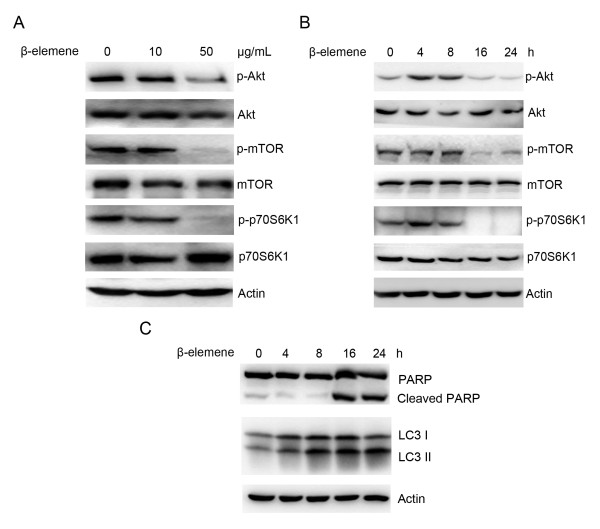
**Effect of β-elemene on the PI3K/Akt/mTOR/p70S6K1 signalling pathway**. (A) MGC803 cells were exposed to 10 or 50 μg/mL β-elemene for 24 h, and the expression of phospho-Akt, phospho-mTOR and phospho-p70S6K1 was detected by western blotting. (B) and (C) Cells were treated with 50 μg/mL β-elemene for the indicated times, and cell lysates were subjected to western blotting with specific antibodies. The results were representatives of three independent experiments. Actin was used as loading control.

It has been well documented that the PI3K/Akt/mTOR/p70S6K1 signalling pathway plays a key role in regulating both apoptosis and autophagy, so the effects of β-elemene on the activity of this pathway were studied next. Cells were treated with 10 or 50 μg/mL β-elemene for 24 h, and the levels of phospho-Akt, phospho-mTOR and phospho-p70S6K1 were detected by western blotting. After treatment with 50 μg/mL β-elemene, the level of phospho-Akt was obviously down-regulated, leading to the down-regulation of downstream phospho-mTOR as well as phospho-p70S6K1 (Figure 4A). A low dose (10 μg/mL) of β-elemene had little effect on PI3K/Akt/mTOR/p70S6K1 activity. Interestingly, a transient activation of PI3K/Akt/mTOR/p70S6K1 was observed after the cells were exposed to β-elemene for short times. As shown in Figure 4B, the level of phospho-Akt increased after 4 h, stayed active until 8 h, and went down after 16 h. Similar changes were observed on the phosphorylation of mTOR and p70S6K1. The cleavage of PARP and conversion of LC3 I to LC3 II was also seen following treatment with 50 μg/mL β-elemene at 16 h and 24 h, consistent with the change of PI3K/Akt/mTOR/p70S6K1 activity (Figure 4C). These data indicated that β-elemene induced apoptosis and autophagy might be due to its inhibition of the PI3K/Akt/mTOR/p70S6K1 signalling pathway.

### β-Elemene-induced autophagy protected MGC803 cells from undergoing apoptosis

**Figure 5:**
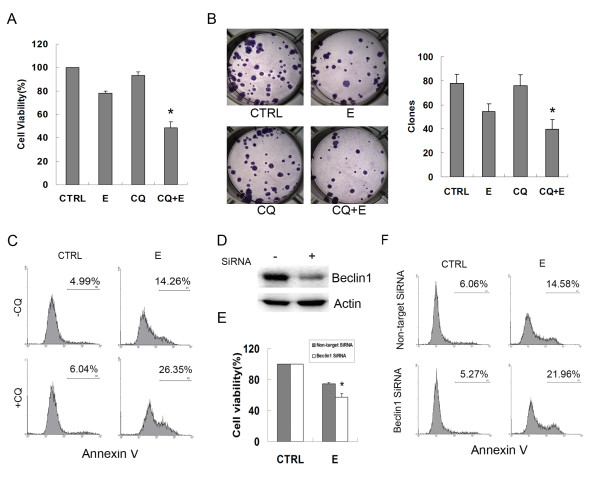
**Inhibition of autophagy enhanced the anti-tumor effect of β-elemene on MGC803 cells**. (A) MGC803 cells were exposed to either 50 μg/mL β-elemene or 20 μmol/L CQ, or a combined treatment of β-elemene and CQ for 24 h, and cell viability was measured by MTT assay. (B) Cells were treated with 50 μg/mL β-elemene or 20 μmol/L CQ, or a combined treatment of β-elemene and CQ for 6 h, and the clone formation ability was analyzed. Columns: mean of three independent experiments; bars: SD. * *P*<0.05 *vs*. cells treated with β-elemene alone. (C) Cells were treated as described in A and apoptosis was analyzed by flow cytometry following Annexin V staning. (D) Cells were transfected with either Beclin1 siRNA or a non-target control siRNA for 48 h, and the expression of Beclin 1 was verified by western blotting. (E) and (F) Forty-eight hours after transfection, the cells were treated with 50 μg/mL β-elemene for another 24 h, and cell viability (E) and apoptosis (F) were analyzed. * *P*<0.05 *vs*. cells transfected with control siRNA.

Since autophagy can result in both survival and cell death, we then asked whether β-elemene-induced autophagy is protective or pro-apoptotic. MGC803 cells were treated with either 50 μg/mL β-elemene or 20 μmol/L CQ (an inhibitor of autophagy which blocks the fusion of autophagosomes with lysosomes, stopping autophagy at the late phase), or co-treated with β-elemene and CQ for 24 h. Cell viability assays showed that co-treatment with β-elemene and CQ significantly decreased cell viability, compared with the cells treated with β-elemene alone (48.50 ± 5.07% *vs*. 78.27 ± 1.57%, *P*<0.05) (Figure 5A). Co-treatment with β-elemene and CQ also significantly reduced the clone formation ability of the cells and increased the apoptotic population compared with the cells treated with β-elemene alone (Figure 5B and 5C). To confirm the effect of autophagy inhibition by the pharmacologic agent CQ on β-elemene-induced apoptosis, an RNA interference approach was used to knock down the expression of Beclin 1. Figure 5D shows that the level of Beclin 1 was significantly decreased in Beclin 1 siRNA-treated cells. Compared with the results in siRNA controls, knockdown of Beclin 1 decreased significantly the cell viability, and enhanced β-elemene-induced apoptosis (Figure 5 E and 5F). These data indicate that blockage of autophagy enhanced the antitumor effect of β-elemene in MGC803 cells.

### β-Elemene induced protective autophagy in SGC7901 gastric cancer cells

**Figure 6:**
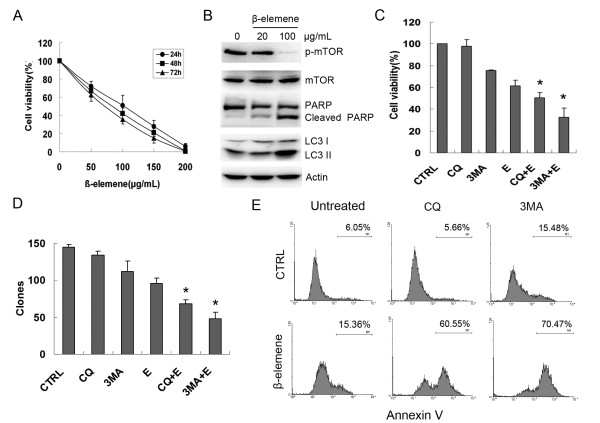
**Anti-tumor effect of β-elemene on SGC7901 gastric cancer cells**. (A) SGC7901 cells were treated with β-elemene at the indicated concentrations for 24, 48 or 72 h, and the cell viability was analyzed by the MTT assay. Dots: mean of three independent experiments; bars: SD. (B) Cells were treated with 20 or 100 μg/mL β-elemene for 24 h, and cell lysates were subjected to western blotting with specific antibodies. (C) Cells were exposed to either 100 μg/mL β-elemene, 10 mmol/L 3-MA, 20 μmol/L CQ, or a combined treatment of β-elemene and 3-MA or CQ for 24 h, and cell viability was measured by the MTT assay. (D) Cells were treated with 100 μg/mL β-elemene, 10 mmol/L 3-MA, 20 μmol/L CQ, or a combined treatment of β-elemene and 3-MA or CQ for 6 h, and the clone formation ability was analyzed. Columns: mean of three independent experiments; bars: SD. * *P*<0.05 *vs*. cells treated with β-elemene alone. (E) Cells were treated as described in A and apoptosis was analyzed by flow cytometry following Annexin V staning.

To prove that the apoptosis and autophagy induced by β-elemene is not cell-specific, we examined the antitumor effect of β-elemene on another human gastric cancer cell line, SGC7901. We found that β-elemene inhibited the viability of SGC7901 cells in a dose-dependent manner, and the IC_50 _values at 24, 48 and 72 h were 89.68 μg/mL, 75.88 μg/mL and 67.13 μg/mL, respectively (Figure 6A). β-Elemene inhibited mTOR activity and induced apoptosis and autophagy, which were evidenced by the cleavage of PARP and the conversion of LC3-I to LC3-II (Figure 6B). The contribution of autophagy to β-elemene-induced apoptosis in SGC7901 cells was evaluated further by co-treating the cells with β-elemene and the autophagy inhibitor, 3-MA or CQ. Compared with the cells treated with β-elemene alone, co-treatment with β-elemene and 3-MA or CQ reduced significantly the viability and clone formation ability of the cells, and increased the apoptotic population (Figure 6C, D and 6E). Similar results from these two human gastric cancer lines indicate that autophagy induced by β-elemene served in a protective manner, and blockage of autophagy enhanced the anti-tumor effect of β-elemene in human gastric cancer cells.

## Discussion

Recently, some traditional Chinese medicines have exhibited promising anti-tumor activity. β-Elemene as a novel anti-cancer herbal medicine has shown broad anti-tumor effects *in vitro *and *in vivo *[2-5]. It has been approved by the State Food and Drug Administration of China for the treatment of malignant effusion and some solid tumors. However, the effects of β-elemene on gastric cancer cells have not been documented. In the present study, we provide the first evidence that β-elemene could inhibit the proliferation of human gastric cancer cells.

Previous study showed that β-elemene induced both G2/M phase arrest and apoptotic cell death in non-small cell lung cancer cells [3]; whereas in ovarian carcinoma cells it only induced cell cycle arrest at G2/M phase [8]. In the present study β-elemene induced obvious apoptosis in gastric cancer cells, but had little effect on cell cycle distribution. This may be due to the different regulating mechanism of cell cycle progress in different types of tumor cells.

Meanwhile, in our study a robust autophagy was observed among the cells treated with β-elemene, which was shown by the increase of punctate LC3 and the morphologic changes. Western blotting also showed a conversion of LC3-I to LC3-II. These specific changes of LC3 have been characterized as an autophagosomal marker in mammalian autophagy. This is the first demonstration that β-elemene could induce autophagy. It has been demonstrated that autophagy is activated by some anti-tumor drugs while they induce apoptosis [14-16]. The autophagy induced by these agents leads to either survival or autophagic cell death. Whether β-elemene-induced autophagy is a protective or a deadly response was confirmed later.

Although the detailed mechanisms regulating autophagy have not been well documented, just like for apoptosis, the process of autophagy is controlled under a group of evolutionarily conserved proteins, the Atg proteins [10]. Accumulated evidence suggest that the induction of autophagy is associated with the up-regulation of certain Atg proteins [21-23]. Thyagarajan et al. reported that triterpenes-induced autophagy is accompanied by the up-regulation of Beclin 1 [21]. Others reported that increased transcription of Atg5 can lead to autophagy [23]. In the present study β-elemene induced autophagy without alternating significantly the levels of Beclin 1, Atg5, Atg9 or Atg16L, while the expression of the Atg5-Atg12 conjugated protein was up-regulated significantly. This is similar to the results reported by Kim et al that irradiation up-regulated Atg5-Atg12 and activated autophagy [24]. Along with the conversion of LC3, the changes of these molecular markers finally lead to autophagy.

The PI3K/Akt pathway has been reported to play an important role in the inhibition of apoptosis [25-28]. Once activated, Akt phosphorylates downstream mTOR, leading to the phospharylation of its target p70S6K1, which promotes cell growth and inhibits apoptosis [29]. Moreover, recent studies have suggested that Survivin is positively regulated by the PI3K/Akt/p70S6K1 pathway [30]. In our study β-elemene inhibited the phospharylation of Akt, mTOR, and p70S6K1, and down-regulated the expression of Survivin but not other apoptotic proteins. This indicated that inhibition of PI3K/Akt/mTOR/p70S6K1 and Survivin by β-elemene might lead to the induction of apoptosis. Meanwhile, mTOR is also a key regulator of autophagy, and inhibition of mTOR activity by some agents has been reported to activate autophagy [31-33]. This may explain the phenomenon of autophagy among the cells treated with β-elemene in the present study.

Since autophagy can result in both survival and cell death, we then investigated whether the autophagy induced by β-elemene was a protective response or a process leading to death. We found that inhibition of autophagy by the autophagy inhibitor, or by genetic knockdown of Beclin 1, the Atg protein essential for autophagy initiation, enhanced significantly the antitumor effect of β-elemene. This phenomenon was also seen in another gastric cancer cell line, SGC7901. These data suggest that the inhibition of PI3K/Akt/mTOR activity by β-elemene resulted in two opposite consequences: on the one hand, it inhibited cell viability and induced apoptosis, which led to death; on the other hand, it activated a protective autophagy to adapt to the stressful conditions and protect cells from death. Inhibition of protective autophagy might be a good way to enhance the anti-tumor effect of β-elemene.

## Conclusions

Taken together, our study provides the first evidence that β-elemene can inhibit the proliferation of human gastric cancer cells by inducing apoptosis. The anti-cancer effect of β-elemene was associated with inhibition of the PI3K/Akt/mTOR/p70S6K1 signaling pathway, which also led to the activation of a protective autophagy. Inhibition of autophagy significantly enhanced the apoptosis-inducing ability, which suggests that the combination of β-elemene with an autophagy inhibitor might be useful for the treatment of advanced gastric cancer.

## Competing interests

The authors declare that they have no competing interests.

## Authors' contributions

JL carried out the cellular and biochemistry study, participated in the transfection and drafted the manuscript. YZ and LX carried out the transfection and participated in fluorescence microscopy assays. JQ and KH carried out the transmission electron microscopy assays. JZ participated in the biochemistry study. XQ and YL conceived of the study, and participated in its design and coordination and helped to draft the manuscript. All authors read and approved the final manuscript.

## Pre-publication history

The pre-publication history for this paper can be accessed here:

http://www.biomedcentral.com/1471-2407/11/183/prepub
